# Current Insights into Sublethal Effects of Pesticides on Insects

**DOI:** 10.3390/ijms25116007

**Published:** 2024-05-30

**Authors:** Merle-Theresa Bartling, Annely Brandt, Henner Hollert, Andreas Vilcinskas

**Affiliations:** 1 Institute for Insect Biotechnology, Justus Liebig University of Giessen, Heinrich-Buff-Ring 26-32, 35392 Giessen, Germany; m.bartling@gmx.net; 2 Bee Institute Kirchhain, Landesbetrieb Landwirtschaft Hessen, Erlenstr. 9, 35274 Kirchhain, Germany; annely.brandt@llh.hessen.de; 3 Department Evolutionary Ecology & Environmental Toxicology, Goethe University Frankfurt, Max-von-Laue-Str. 13, 60438 Frankfurt am Main, Germany; hollert@bio.uni-frankfurt.de; 4 Department Environmental Media Related Ecotoxicology, Fraunhofer Institute for Molecular Biology and Applied Ecology, Auf dem Aberg 1, 57392 Schmallenberg, Germany; 5 LOEWE Centre for Translational Biodiversity Genomics (LOEWE-TBG), Senckenberganlage 25, 60325 Frankfurt, Germany; 6 Branch of Bioresources, Fraunhofer Institute for Molecular Biology and Applied Ecology, Ohlebergsweg 12, 35392 Giessen, Germany

**Keywords:** beneficial arthropods, insecticides, pesticides, pest insects, sublethal doses, toxicology

## Abstract

The effect of pesticides on insects is often discussed in terms of acute and chronic toxicity, but an important and often overlooked aspect is the impact of sublethal doses on insect physiology and behavior. Pesticides can influence various physiological parameters of insects, including the innate immune system, development, and reproduction, through a combination of direct effects on specific exposed tissues and the modification of behaviors that contribute to health and reproductive success. Such behaviors include mobility, feeding, oviposition, navigation, and the ability to detect pheromones. Pesticides also have a profound effect on insect learning and memory. The precise effects depend on many different factors, including the insect species, age, sex, caste, physiological condition, as well as the type and concentration of the active ingredients and the exposure route. More studies are needed to assess the effects of different active ingredients (and combinations thereof) on a wider range of species to understand how sublethal doses of pesticides can contribute to insect decline. This review reflects our current knowledge about sublethal effects of pesticides on insects and advancements in the development of innovative methods to detect them.

## 1. Introduction 

Declining insect populations have been widely reported in the scientific literature and mainstream media [1,2], although the true extent of the phenomenon on a global scale is unclear because the various studies are localized and often focus on “popular” insects such as butterflies and bees [3]. Reviews based on data from long-term population surveys [4,5] have been criticized for methodological limitations, including geographic bias, species bias, and the selection of confirmatory reports [6,7,8]. Some studies have found alarming declines in insect abundance, biomass or biodiversity over time [9,10], whereas others have reported no significant overall change [11]. More detailed analysis is clearly necessary [12].

**Figure 1:**
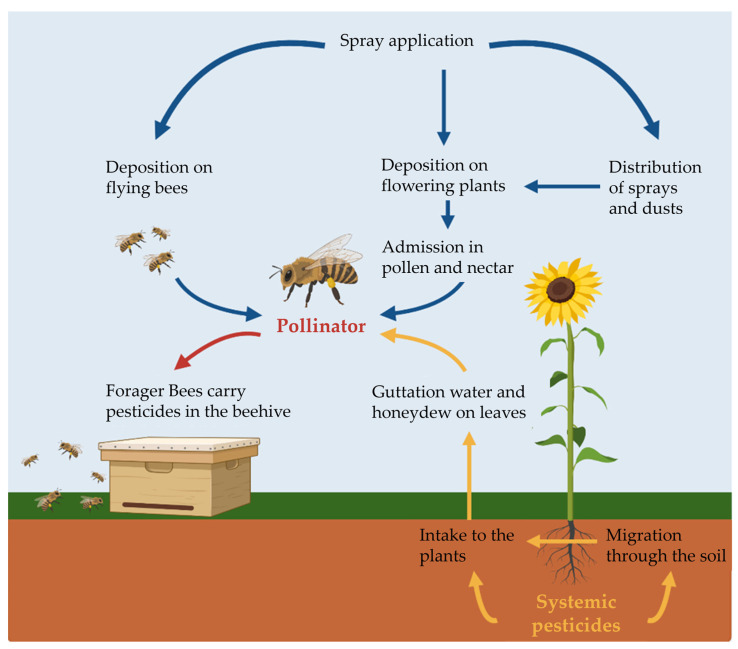
Summary of possible exposure routes of plant protection products applied by spraying and systemic pesticides using the example of honeybees (according to IPBES 2016, created in biorender.com).

Despite many reports confirming insect population decline, at least in a local context, it has not been possible to attribute a specific cause, leading to the multi-causal hypothesis. The various contributory factors are proposed to include habitat loss, agricultural monocultures, climate change, the spread of diseases and parasites [13], and the use of pesticides. Currently, more than 3 million tons of pesticides costing USD 40 billion are applied annually to crops [14,15], with the total amount almost doubling between 1990 and 2019 [16]. There are many ways in which insects can come into contact with pesticides (Figure 1). For example, bees can take up pesticides in their food, through direct contact with nesting materials and other contaminated surfaces, through inhalation and exposure to water containing dissolved pesticides, and through indirect contact as contaminated materials are distributed within the hive [17].

**Figure 2:**
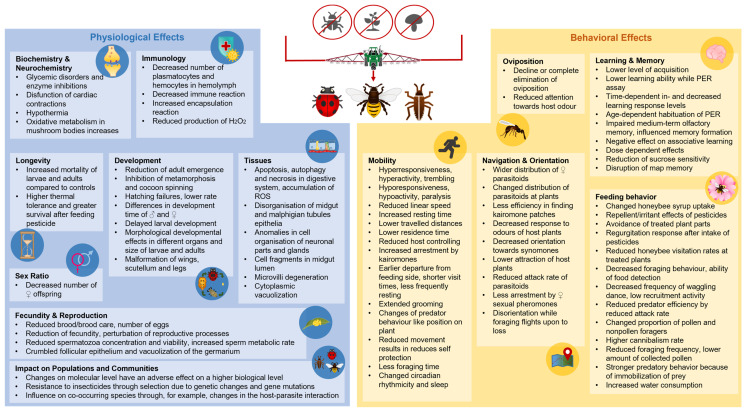
Overview of the various physiological and behavioral effects of sublethal amounts of pesticides on insects. The impacts are divided into physiological effects and behavioral effects. For these two groups, they are further divided into biochemistry and neurochemistry [21,22,23,24,25], immunology [26,27,28,29,30,31,32,33,34,35,36,37,38,39,40,41], longevity [30,35,41,42,43,44,45,46,47,48,49,50,51,52,53,54,55,56,57,58,59,60,61,62,63,64,65,66,67,68,69,70,71,72,73], development [26,41,49,50,51,52,56,57,58,59,60,61,62,63,64,65,66,67,68,69], tissue [74,75,76,77,78,79,80,81,82,83], fecundity & reproduction [30,44,54,55,59,61,68,84,85,86,87,88,89,90,91,92,93], Impact on Populations and Communities [94,95,96,97,98,99], sex ratio [88,89,90], mobility [74,75,100,101,102,103,104,105,106,107,108,109,110,111], egg laying [47,112,113,114,115,116,117], navigation & orientation [17,107,109,110,116,118,119,120,121,122,123,124,125,126,127,128,129,130,131,132], learning & memory [25,133,134,135,136,137,138,139,140,141,142,143,144,145,146,147] and feeding behavior [56,74,118,133,134,135,148,149,150,151,152,153,154,155,156,157,158,159,160,161,162,163,164,165,166,167,168,169].

The lethal effects of pesticides are often categorized as acute or chronic [18]. Acute toxicity results in death within 72 h, whereas chronic toxicity takes longer and often reflects the intake of sublethal quantities that accumulate in the body [19]. However, sublethal doses of pesticides can have a profound physiological and behavioral effect even if the insect survives [17]. This aspect has been neglected in the literature and is addressed in this review. A large number of active ingredients and their combinations show a wide variety of effects, but these have not been investigated for 71% of individual substances and 99% of their combinations [20]. A bibliometric analysis in SCOPUS (18.5.2024) with the key words pesticides, sublethal, and insects resulted in 724 documents. This review summarizes recent research concerning the sublethal effects of pesticides on insects, considering the impact on physiology and behavior (Figure 2).

## 2. Physiological Effects 

### 2.1. Biochemistry and Neurochemistry

Sublethal doses of pesticides often affect the biochemistry and neurochemistry of insects due to the inhibition of enzymes and signaling proteins, with consequential effects on physiology [21]. For example, in *Apis mellifera* (Linnaeus, 1758), deltamethrin or prochloraz increase the metabolic rate, leading to hyperthermia [22] and heart arrhythmia [23,24]. The neonicotinoid imidacloprid was found to stimulate oxidative metabolism in the mushroom bodies of the honey bee brain, affecting medium-term olfactory memory [25].

### 2.2. Immunology

Possible interactions between pesticides and the immune system of insects have been investigated for mainly two reasons. First, to improve pest control strategies, e.g., to determine whether the activity of biological pesticides can be enhanced with certain chemical pesticides [26]. Second, to investigate whether sublethal dosages of pesticides might render non-target insects more susceptible to diseases, especially beneficial insect pollinators like bees [26]. 

Insect immunity is basically composed of three parts: the cuticle, which presents physical and chemical barriers, the humoral immune responses, and cellular responses mediated by hemocytes. Pesticides often trigger a general suppression of the insect immune response. For example, *A. mellifera* workers and queens exposed to sublethal dosages of the neonicotinoids thiacloprid, or thiamethoxam, showed a reduced number of hemocytes, pronounced inhibition of the encapsulation response, and reduced antimicrobial activity [27,28]. The sublethal effects of pesticides cannot be generalized to other species or different sexes. In the solitary bee species *Osmia bicornis* (Linnaeus, 1758), thiacloprid reduced the number of hemocytes only in males, but not in females, whereas the antimicrobial activity was not affected [29].

In the bug insect *Rhynocoris kumarii* (Ambrose and Livingstone, 1986), the oral administration of monocrotophos, methyl parathion and endosulfan specifically reduced the number of plasma cells in the hemolymph, reflecting the conversion of plasmocytes into granular hemocytes during pesticide detoxification [30]. For example, the insecticide dieldrin suppressed the activation of defense mechanisms against parasitic wasps by 25% in the fruit fly *Drosophila melanogaster* (Meigen, 1830) [31]. Organophosphates inhibit the proliferation and differentiation of honey bee hemocytes, suppressing phagocytosis, melanization and the phenol oxidase cascade [26]. Organophosphates also inhibit the production of hydrogen peroxide (H_2_O_2_), which is used as a signaling molecule in *D. melnogaster* and a primary response against pathogens [32]. In contrast, chlorpyrifos promoted the encapsulation reaction in the parasitoid wasp *Leptopilina boulardi* (Carton and Keiner-Pillault, 1979) [33]. The herbicide glyphosate was shown to repress honey bee genes responsible for detoxification such as *CYP9Q2*, *CYP6AS4* and *CYP9Q3*, genes encoding the enzymes pacifastin, metalloprotein—MME, lysozyme, glucose oxidase and vitellogenin, genes involved in plant–herbivore interactions (G12-like protein), and genes such as *GB46620* controlling epigenetic mechanisms [34,35,36]. More recent studies show that the redox reactions needed for melanization are also disrupted by this herbicide [37]. The suppression of these natural immunity mechanisms makes the bees more vulnerable to the effects of pesticides [38] and oxidative stress in general [35,39]. The influence of pesticides on epigenetic control mechanisms resulted in heritable effects that were observed in subsequent generations [40,41]. 

### 2.3. Tissues

Pesticide-induced changes in specific tissues have been reported, especially in areas of direct contact with active substances such as the body surface, the intestinal epithelium, and the epithelium of the Malpighian tubules [74]. Sublethal effects include the disorganization of the microvilli in the gut of the crop pest *Anticarsia gemmatalis* (Hübner, 1818) [75], as well as the induction of apoptosis, autophagy, and necrosis in the digestive system and the accumulation of reactive oxygen species (ROS) in the intestine, often leading to detoxification responses [74,75,76,77]. Cell fragments were found in gut lumen of *A. mellifera* after feeding with spiromesifen [78] and lambda-cyhalothrin [79]. Sublethal doses of pesticides are also associated with larger gaps in the neuropil of the brain and mushroom bodies (with a knock-on effect on lifespan and behavior) as well as cytoplasmic vacuolization and cell death [77,80,81,82,83].

### 2.4. Longevity and Development

Most studies considering the effect of pesticides on longevity have reported higher mortality in larvae and/or adults, as would be expected [42,43,44,45,46,47,48,49,50]. However, at least one study has shown that *A. mellifera* exposed to low levels of pesticides are more likely to survive heat stress, with survival rates increasing by up to 87% [51]. Pesticides can also affect insect development. Following exposure to spinosad and fenoxycarb, the parasitic wasp *Hyposoter didymator* (Thunberg, 1822) and the lacewing *Chrysoperla carnea* (Thunberg, 1822) showed severe limitations in their ability to spin cocoons, in some cases including the complete absence of the silk production [52,53]. Various pesticides were shown to accelerate the development of the parasitic wasp *Trichogamma pretiosum* (Riley, 1879), ultimately inhibiting its capacity for parasitism [54], whereas bifenthrin and deltamethrin promote maturation in *A. mellifera*, resulting in a lower hatch rate [55], and imidacloprid with or without lambda-cyhalothrin inhibits honey bee worker development and brood number [56]. The genes modulated by glyphosate (see above) also include several needed for *A. mellifera* growth and development [57]. Honey bees fed for several days on glyphosate, which was brought into the beehive by forager bees (Figure 1), failed to develop normally, including incomplete and unsuccessful molts [35]. In the insect species *Supputius cincticeps* (Distant, 1889), permethrin was shown to accelerate female development but delay male development [58]. Indeed, sublethal doses of many pesticides are known to inhibit larval growth, such as sulfoxaflor in the common bumble bee (*Bombus terrestris*, Linnaeus, 1758) and thiamethoxam in the lacewing *Chrysoperla externa* (Hagen 1861) and the harlequin ladybird *Harmonia axyridis* (Pallas, 1773) [59,60]. The effects on development are pesticide-specific and species-dependent, but may also be influenced by the study method, the physiological condition of the test insects and the period of testing [17]. For example, imidacloprid did not influence the development time of *B. terrestris* bee larvae, in contrast to the previously reported treatment with sulfoxaflor [61]. Overall, fewer broods are reported after pesticide treatment [62], along with changes in size, weight, and morphology compared to controls [30,63,64,65,66,67,68,69,70,71]. The development of different castes in eusocial insects is also epigenetically regulated [72,73], so the influence of pesticides on epigenetic mechanisms can affect the entire community [41].

### 2.5. Fecundity and Reproduction

The most common reproductive effects of sublethal pesticides include reduced fecundity [54,55], a lower egg-laying rate, or a reduced amount of brood in general [44,61,84,85]. The number of eggs laid may fall by up to 45% over several generations [59,68]. For example, fipronil reduces the concentration and availability of spermatozoa in drones of *A. mellifera* and thus limits male fertility [86]. The same effect was observed in *Tribolium castaneum* (Herbst, 1797) following the administration of two different drugs, and was passed to the next generation due to epigenetic regulation [87]. However, pesticides also cause morphological changes in the reproductive system. The botanical azadirachtin causes ovarian atrophy in the bumble bee, with higher concentrations resulting in the absence of oocytes [44]. Other effects include a crumbled follicular epithelium and vacuolization of the germarium in the ovaries of the assassin bug *R. kumarii* [30]. A shift in the sex ratio towards more male offspring could be shown in various parasitoid wasps [88,89,90]. Using in-hive video recordings, reduced feeding visits and feeding durations of nurse bees in response to retarded larval development in clothianidin or thiacloprid-exposed *A. mellifera* colonies could be shown [91]. Moreover, in *A. mellifera*, exposure to sublethal doses of clothianidin caused workers to change the composition and production of royal jelly, which did not change the number of encapsulated brood cells but did increase larval mortality [92]. Honey bees, thus, appear to compensate for pesticide-induced brood death by increasing the brood initiation rate. Computer simulations predicted that these compensation mechanisms weaken the colony by inhibiting swarming behavior and reducing long-term survival. In *A. mellifera*, exposure to the neonicotinoids thiamethoxam and clothianidin can also affect the reproductive capacities of males. Although the total number of drones produced by a colony was not altered, the drones delayed flight activities by 3 days, and the number of living sperm was reduced by 28% [93].

### 2.6. Impact on Populations and Communities 

The effects of sublethal doses of pesticides on insects at the level of populations or communities have so far received little scientific research. The complexity of the question represents an obstacle, as long-term effects must also be taken into account, including from pesticide mixtures, degradation products or interactions between the ecosystem and the active ingredient. For example, it may be that single individuals are influenced, which results in a change in conspecific interactions and can even trigger population-level responses (e.g., insecticide resistance and control failure), which in turn can be transferred to the level of entire communities [94]. This process is often described under the concept of the adverse-outcome pathway. This considers how a change on a molecular level can have an adverse effect on a higher biological level in order to arrive at a risk assessment [95,96].

A change in resistance and mortalities to insecticides caused by selection due to genetic changes can then in turn influence the success of pest control in the field [97]. It has been observed that sublethal doses of pesticides promote polygenic resistance or gene mutations involved in DNA repair in the surviving organisms [98]. In addition, the resistance of insects to pesticides can result in insecticide-induced hormesis or induction/cross-induction of detoxification enzymes. The application of pesticides can therefore lead to an increased growth of the resistant population.

Heterospecific interactions between co-occurring species can also be influenced. It is possible, for example, that host-parasite interactions can be disrupted, the dominance of individual species can be shifted, or species interactions can be changed. All of this can result in insecticide-induced community stress [98].

## 3. Behavioral Effects

The awareness that contaminants and other environmental stressors have a significant influence on the behavior of organisms and can therefore also have negative effects on entire ecosystems is growing increasingly with new research findings [99]. However, there are still some gaps in knowledge regarding behavioral ecotoxicology, which then affect the assessment of risks from pesticide use.

Behaviors determine, among other things, individual fitness by influencing reproductive success, which has consequences for population dynamics, species interactions, and ecosystem function [99]. The complexity of all interactions and processes that can be influenced, and the resulting far-reaching consequences, show the importance of considering this topic, for example when assessing the risk of pesticides.

### 3.1. Mobility

Sublethal doses of pesticides have been shown to enhance or inhibit mobility depending on the dose, context and timing. For example, *A. mellifera* fed on low doses of imidacloprid, and its metabolites initially showed a period of hyperactivity and trembling followed by sluggish activity [100]. However, a high dose (41 ng/bee) resulted in immediate paralysis, which resolved within 48 h as the pesticide was degraded [101]. Paralysis and trembling are also caused by sublethal doses of other pesticides, again depending on the dose [102,103]. Exposure to pesticides has in some cases been shown to extend the resting time of insects [74,75,104,105], but in other cases, the resting time was shorter [106,107,108]. Some plant protection agents appear to reduce the speed of insect movement, whereas spinosad has the opposite effect [75,105,107]. 

Pesticides also stimulate grooming behavior in many insects, perhaps because they act as irritants. Contact with deltamethrin has this effect on the seven-spotted ladybird *Coccinella septempunctata* (Linnaeus, 1758) and the wasp *Aphidius rhopalosiphi* (de Stefani-Perez, 1902) [106,109,110]. Deltamethrin leading to *C. septempunctata* not only results in significantly higher grooming, but also significantly lower resting. Treated ladybird beetles moved closer to the ground on the plant more often than controls. Exposed *A. rhopalosiphi* showed shorter retention times, shorter visit times at feeding places, less imposed resting, and extended grooming than controls. The cockroach *Periplaneta americana* (Linnaeus, 1758) and the ground beetle *Harpalus pennsylvanicus* (DeGeer, 1774) also show this reaction [102,111]. 

### 3.2. Feeding Behavior

The desired effects of pesticides include the reduction of feeding damage by crop pests [148], but a similar impact is observed in non-target insects, such as reducing the foraging time, frequency, and efficiency in honey bees and bumble bees [56,74,133,149]. Pesticides can accumulate in all plant tissues and secretions, including pollen and nectar, and pollinators are therefore quickly exposed to the active substances [150]. The rate of honey bee visits to feeding sites decreased significantly in one study when the syrup was treated with fipronil [151], whereas fipronil and imidacloprid did not affect frequency of visits in another study, but did minimize food intake and feeder activity [152]. Similarly, the oral intake of deltamethrin reduced syrup uptake by honey bees and bumble bees [153]. Thiamethoxam caused bumble bees to engage in longer foraging bouts with less frequent returns to the nest to deliver the pollen [118]. For other pesticides, such as the botanical azadirachtin, no change in the rate of visits to feeding stations was observed [154]. Similarly, no change in foraging activity was observed when bees were exposed to pollen contaminated with the fungicides chlorothalonil and propicanizole or the insecticides chlorypyrifos and fenpropathrin [155]. However, waggle dancing by *A. mellifera* was inhibited by imidacloprid, thus reducing recruitment activity and food procurement [156]. In some predatory carabid species such as *Pterostichus melas* (Creutzer, 1799) and *Poecilus koyi* (Germar, 1823), acute thiamthoxam intoxication also leads to lower food intake [157].

The topical application of fipronil to the thorax of *A. mellifera* made their antennae less sensitive to low concentrations of sucrose, inhibiting their ability to track food sources [134]. Interestingly, exposure to thiamethoxam causes very young worker bees to take over the function of foragers and commence foraging flights, increasing the risk of forager bee loss [158]. Pesticides such as fipronil and acetamiprid also increase the thirst response of honey bees, resulting in the uptake of more water [135]. 

Sublethal pesticides also reduce predation rates by up to 90% [159]. This was observed after exposure of *Serangium japonicum* (Chapin, 1940), a biological agent for the control of *Bemisia tabaci* (Gennadius, 1889), to thiamethoxam [160]. However, the predation rate of *S. japonicum* recovered after 24 h of exposure or 24 h after the end of exposure. Larvae of *C. septempunctata* consume fewer aphids on plants treated with deltamethrin, which may reflect avoidance behavior [159,160,161]. If the aphids themselves are treated with azadirachtin, a combined repellent and antifeedant effect is observed in ladybirds and lacewings [163]. In contrast, the 11-spotted ladybird (*Coccinella undepunctata* Linnaeus, 1758) became more predacious following exposure to pirimicarb and pymetrozine, but this was an indirect effect caused by the reduced mobility of prey [164]. On the other hand, permethrin, tebufenozide and thiamethoxam have a direct repellent effect on the predatory bug species *Podisus nigrispinus* (Dallas, 1851) [165]. The attack rate of predatory species is often reported to decline in the presence of insecticides, and the prey handling time increases [166,167]. The process of catching prey and, for example, the ability of the assassin bug *Acanthaspis pedestri* (Stål, 1863) to paralyze its prey, can also be inhibited [166]. The species *A. pedestri* also showed concentration-dependent random movement and restlessness following treatment with cypermethrin. Likewise, the beetle *Nebria brevicollis* (Fabricius, 1792) regurgitated 53–80% of aphids treated with deltamethrin [168]. A change in the feeding rate or the amount of food consumed can also influence food–gene interactions, as previously shown with broccoli and stress-resistance genes in *T. castaneum* [169]. Pesticides ingested with the feed also can influence gene expression, as discussed above.

### 3.3. Oviposition Behavior

The oviposition of many insects is affected by sublethal levels of pesticides. One study reported that the frequency of oviposition into host larvae by the parasitoid wasp *Leptopilina heterotoma* (Thomson, 1862) increases by up to 46% in the presence of chlorpyrifos [112]. However, most reports provide evidence of the opposite effect, with less frequent oviposition and fewer sting attacks, sometimes even with the complete cessation of oviposition activity [113,114,115,116,117]. The effects are highly dependent on the specific active ingredient. For example, exposing *Trichogramma achaeae* (Nagaraja & Nagarkatti, 1970) to chlorantraniliprole, pirimicarb and azoxystrobin resulted in a constant or increased rate of parasitism, whereas methiocarb, spinosad and chlorpyrifos inhibited or even completely abolished this activity [47]. Some parasitoids even leave plants when they are treated, reducing the amount of aphid parasitism [110]. Scent-mediated host recognition and search behavior by parasitoids can also be inhibited by pesticides [52].

### 3.4. Navigation and Orientation

The ability of insects to navigate and orientate is also affected by sublethal levels of pesticides. For example, deltamethrin caused females of the parasitic wasp *A. rhopalosiphi* to disperse widely. They spent less time in one spot and appeared restless, resulting in a lower rate of predation [109,119]. On the other hand, females of the parasitoid wasp *L. boulardi* that came into contact with the insecticide chlorpyrifos rested for longer and were less efficient in the detection of kairomone patches, resulting in lower fecundity [120]. If a host patch was found, the parasitoid residence time was shorter [107]. When honeydew was mixed with deltamethrin, *A. rhopalosiphi* departed from the patches much earlier than controls that were not exposed to the insecticide [109]. In another *Aphidus* species, attraction and orientation towards host-plant odors were reduced by up to 71% following exposure to pesticides [116,121,122]. Pesticides can also change the part of the plant on which parasitoids reside [110]. 

The oral intake of fipronil, clothianidin, thiacloprid, imidacloprid and other pesticides causes a decline in the orientation of honey bees, for example landmark use [123,124,125,126], sometimes with fatal consequences [127]. Neonicotinoids in particular inhibit the homing ability of bees [17]. In one study, bumble bees were better able to find their nest 1 km away following exposure to thiamethoxam [118], possibly reflecting the longer orientation flights of the pesticide-exposed group. However, imidacloprid reduced the flight distance and duration to around a third, with increased velocity [128]. This explains the known negative effects of pesticides on pollination service capabilities, with a lower abundance, diversity and nutritional quality of food. 

Sublethal quantities of pesticides also have an effect on the orientation of parasitoids towards sexual pheromones. Males of the parasitic wasp *Trichogamma brassicae* (Bezdenko, 1968), an important biological control species, showed less interest in female pheromones when exposed to chlorpyrifos, and females also produced lower levels of pheromones [129,130]. Small amounts of deltamethrin caused males to show more interest in female pheromones, but the scent of treated females was less attractive [131]. Clothianidin had no effect on the orientation of monarch butterfly (*Danaus plexippus*, Linnaeus, 1758) caterpillars [132].

### 3.5. Learning Behavior

Many olfactory conditioning experiments have been carried out using different bee species, and the success of learning in the presence and absence of pesticides has been queried using the proboscis extension response (PER). The effects were dependent on the active substance, its concentration, the insect species, and also the age and sex of the test subjects. However, all the pesticides caused a reduction in olfactory learning and/or memory performance. Feeding a sugar solution spiked with deltamethrin, endosulfan, prochloraz or fipronil resulted in significantly lower responses during PER assays [136]. The acaricide diazinon also inhibits learning in *A. mellifera*, whereas coumaphos has a lesser effect [137]. Imidacloprid not only inhibits the learning process, but also interferes with medium-term olfactory memory, which reflects the disruption of transfer from short-term to medium-term memory [25,133]. This effect was age-dependent, with learning inhibited in bees that were 7 days old or 9+ days old but improved in bees that were 8 days old [138]. A similar course was observed when feeding with permethrin [139]. Imidacloprid also induces significant habituation of the PER in honey bees [140]. 

In our own studies, we were able to show that the neonicotinoid clothianidin also had negative effects on the learning behavior of *A. mellifera* at concentrations more than ten times lower than the LD50 specified by the manufacturer [141]. This was carried out using the APIS conditioning chamber, a test system in which the ability to learn scents is tested in an aversive procedure, excluding surrounding stimuli. Aversive conditioning was chosen because it is considered more efficient than traditional PER conditioning [142]. Since the assessment of learning success is based on the movement pattern of the animals, a test was then carried out to determine whether a change in movement intensity could be ruled out by feeding clothianidin. No significant difference could be found in the general movement patterns between control and pesticide bees. This suggests that the result observed in the conditioning chamber is due to an altered learning ability. The insecticide flupyradifurone, which has a similar effect to neonicotinoids, also impaired learning by 48%/74% and memory by 22%/48% in adult bees/larvae [143,144]. Imidacloprid metabolites also have a negative impact on bees, and the effects of 5-OH-imidacloprid are five times more potent than the parent substance [145]. A definitive statement about the effects of a pesticide is often difficult to make due to various factors that come into play during testing. For example, it could be shown that chronic feeding of doses of the fungicide amistar, which are considered non-hazardous, causes a negative trend in the formation of memory in bumble bees [146]. For more appropriate legislation regarding the use of pesticides, more understanding of the mode of action and residue levels of active ingredients must be gained. The studies described above involved the oral intake of pesticides, but even contact with a pesticide-contaminated surface can reduce learning ability and antennal sucrose stimulation during the PER tests in *A. mellifera*, as shown for small doses of thiamethoxam and fipronil [134,135]. Interestingly, insecticides appear to have a greater effect in bees of the genus *Apis* than those of the genus *Bombus* [147].

## 4. Synergistic Interactions

Individual pesticides have been developed in order to control agricultural pest insects and vectors of human diseases, and have been optimized to prevent the mortality in beneficial insects. Hence, it is well known that one pesticide may render the insect organism more sensitive to a second stressor and that environmental pollutants may interact [170]. A pesticide, which is regulated on a single compound basis and deemed non-harmful for beneficial insects, can be potentiated by other chemicals, so that they jointly exert a larger effect than predicted for the individual substances. An example of this is the herbicide glyphosate [171]. These synergistic interactions of pesticides in mixtures are an area of great concern to both the public and regulatory authorities in Europe and the US [172]. Yet, current laws do not require risk assessments of combinations for pesticides on the market, except in very limited instances [173].

Synergistic effects occur when combined exposure to two or more factors results in an effect that is significantly greater than the sum of the individual effects [174]. Such synergistic effects can occur between pesticide and poor nutrition [175], or between pesticides and diseases [176], or from exposure to multiple pesticides [177]. The combination of diseases by fungi and pesticide (imidacloprid) can synergistically alter beetle movement [178]. A prominent example for field-relevant synergistic interactions of pesticides is the interaction between neonicotinoids and SBI (sterol biosynthesis inhibitor) fungicides on honey bees, because SBI fungicides can inhibit detoxification in insects [179] and affect the behavior of honey bees (poor coordination, hyperactivity, apathy; [173]). A recent study shows that the combination of the fungicide Sakura^®^ and the herbicide Elegant 2FD resulted in neurotoxic effects and induced detoxification processes in honey bees. Further, exposure to the herbicide/fungicide mixture impaired their learning and memory [180].

## 5. Future Prospects

Insects cause enormous yield losses globally during agricultural production and storage and can, therefore, be considered as the most important competitors for human nutrition. The food supply security for the growing human population depends on industrial agriculture encompassing the use of pesticides. Lethal concentrations of insecticides trigger the evolution of resistance by strong directional selection. A recent study demonstrates that also transgenerational sublethal exposure to an insecticide can raise resistance in the offspring of the Colorado potato beetle (*Leptinotarsa decemlineata*, Say, 1824) via undesired positive hormetic effects [181]. Our current insights into sublethal effects of pesticides on insects argue for extended and ambitious efforts to reduce the health risk for them and other nontarget organisms [12,182]. In this context, the EU, for example, plans further restrictions for the approval of novel pesticides. The risk assessment for the latter should be expanded beyond the existing guidelines to encompass innovative methods allowing the detection of harmful influences on complex parameters such as behavior, fecundity, longevity, learning, and memory in insects, particularly in honey bees and other pollinators. However, this results in a dilemma because extended risk assessment and safety hurdles increase the costs for the development of novel pesticides enabling the future control of pest insects that evolved resistance to those that are currently used. If the costs for the development of new pesticides exceed the expected return on investment, even big agricultural companies will not further invest in chemical plant protection. In order to replace successively chemical pesticides, we need increasing investment in alternative, more sustainable and species-specific control options for pest insects such as those based on RNA interference or biological control agents.

## 6. Conclusions

The majority of studies addressing the impact of pesticides on beneficial insects focus on honey bees and other pollinators. The sublethal effect of pesticides on non-target insects depend on factors such as the insect species, age, sex, caste, physiological condition, as well as the type and concentration of the active ingredient(s) and the exposure route. Both positive and negative effects have been observed, although negative effects are more commonly reported. It can also be difficult to reach a precise definition of a sublethal dose. For example, the moment of death in *A. mellifera* is difficult to determine because this would require the measurement of vital signs such as heartbeat, respiration, or neural activity. However, honey bees can recover from a completely immobile and inert state, which according to OECD guidelines is indicative of death [101]. In order to assess the importance of sublethal amounts of pesticides in relation to insect decline, it is important to study the effects of different doses and exposure routes in a broad range of species, and also to study the impact of combinations of different active ingredients [12,180,181,182].

## Data Availability

Not applicable.
